# Toward industry 5.0: Challenges and enablers of intelligent manufacturing technology implementation under the perspective of sustainability

**DOI:** 10.1016/j.heliyon.2024.e35162

**Published:** 2024-07-25

**Authors:** Shiyan Liu, Pengyue Li, Jinfeng Wang, Peng Liu

**Affiliations:** a School of Management, Zhengzhou University, Zhengzhou 450001, China; b China Institute of FTZ Supply Chain, Shanghai Maritime University, Shanghai 201306, China

**Keywords:** Industry 5.0, Intelligent manufacturing technology, Sustainable development, Adversarial interpretive structural modeling (AISM), Matrix impacts cross-reference multiplication applied to a classification (MICMAC)

## Abstract

The advancement of intelligent manufacturing technology in the era of Industry 5.0 has propelled the intelligence and automation of manufacturing production, while also exerting a significant impact on sustainable development of the manufacturing industry. However, the challenges and enablers faced by the transformation of intelligent manufacturing technology in the context of sustainable development of Industry 5.0 are still unclear. Based on literature review and expert opinions, this study uses the Likert scale to determine the challenges and enablers of the implementation of intelligent manufacturing technology in social, environmental and economic sustainability. The fuzzy-DEMETAL and AISM are used to analyze the logical relationship and hierarchical relationship between the above factors, and the MICMAC matrix is used to determine the key influencing factors. The research conclusions show that the most important challenges affecting the implementation of intelligent manufacturing technology are cost and funding, and the most important enabler is social benefits and public service improved. This research will provide insights for industry practitioners and decision makers in the management and decision-making process of implementing the transformation and upgrading of manufacturing intelligent manufacturing, thereby enhancing the sustainability of manufacturing development.

## 1 Introduction

Intelligent manufacturing aims to connect and integrate existing resources and use modern technology to improve productivity and efficiency [1]. Since the concept of intelligent manufacturing was first mentioned in the era of Industry 4.0 [2,3], the concept of economic development for the purpose of improving production efficiency is consistent with the optimization standard of traditional business model, so intelligent manufacturing technology has developed rapidly under the impetus of global disruptive technology. Emerging technologies such as cyber-physical systems (CPS), artificial intelligence, big data, industrial interconnection and industrial Internet of Things have been continuously integrated into the continuous transformation and upgrading of the manufacturing industry, promoting automation and intelligence in various fields of the value chain and reshaping the production mode of industrial systems. As an important part of Industry 4.0, intelligent manufacturing is helping the manufacturing industry gradually realize a highly interconnected network physical manufacturing system [4].

Although the arrival of Industry 4.0 emphasizes productivity and efficiency, it is accompanied by a significant increase in economic and social problems such as global climate change, resource shortages, and the COVID-19 pandemic [5]. Obviously, the conceptual model of Industry 4.0 aimed at maximizing productivity does not conform to the current concept and trend of sustainable development, especially from the perspective of social sustainability and environmental sustainability. At the same time, in order to continuously promote the practice of industrial transformation and begin to build a more flexible and resilient economic system, with the deepening of exploration and the continuous accumulation of experience, a new paradigm to guide the development of manufacturing industry-Industry 5.0 came into being [6].

Industry 5.0 is the deepening and expansion of the connotation and outer edge of Industry 4.0. The most significant change is to focus more on the social and environmental benefits of manufacturing development [7]. Compared with the technology-centered concept of Industry 4.0, Industry 5.0 aims to promote the further transformation of manufacturing production mode and technology development trend to resilience, sustainability and people-oriented [8].

Obviously, the application of Industry 5.0 will bring technological subversion, excellent performance and sustainable development to the manufacturing industry [3,7]. As the core technology of manufacturing transformation and upgrading, intelligent manufacturing integrates information technology, advanced manufacturing technology, automation technology, artificial intelligence technology and so on. It is bound to be given new meaning in the development of industrial 5.0 paradigm, emphasizing the cooperation between human and machine, and more emphasis on sustainability and flexibility. However, under the background of the rapid development of information technology and the guidance of the concept of sustainable development, how to realize the sustainable development, transformation and upgrading of manufacturing industry has become an important issue concerned by the state and enterprises.

In view of this, in order to ensure that the manufacturing industry successfully implements intelligent manufacturing technology in the context of industry 5.0 and sustainable development, and realizes the continuous transformation and upgrading of the manufacturing industry. It is necessary to identify and evaluate the main challenges and enablers in the implementation of intelligent manufacturing technology. Although many studies have emphasized the importance and impact of Industry 5.0 on sustainable development and industrial development [[9], [10]], some scholars have discussed and evaluated the key enabling technologies of Industry 5.0 [3]. However, the current research on industry 5.0 and sustainable development is less involved, and there is still little understanding of the implementation of intelligent manufacturing technology in the manufacturing industry to achieve industry innovation and the interaction between social, environmental and economic sustainable development. With reference to the above research gaps, this study explores the answers to the following research questions.

RQ1 From the perspective of Industry 5.0 and sustainable development, what are the main challenges and enablers of implementing intelligent manufacturing technology in the manufacturing industry?

RQ2 How to evaluate these challenges and enablement to improve the sustainability and performance of intelligent manufacturing technology in manufacturing applications?

RQ3 What is the relationship and importance between identified challenges and enablers?

In order to solve the above problems, this study based on the proposed framework of the social, environmental and economic sustainable development perspective of Industry 5.0, systematically understands the challenges and empowerment of manufacturing industry to implement intelligent manufacturing technology. The innovation and contribution of this study are as follows.

(1) This paper systematically clarifies the challenges and enablers of implementing intelligent manufacturing technologies under the background of Industry 5.0, and the background of social sustainability, economic sustainability, and environmental sustainability by using fuzzy linguistic sets.
(2) This paper analyzes the importance and logical hierarchical relationship of obstacles or driving factors under the background of economic, environmental, and social sustainability development, revealing the relevance and importance between challenges and enablers.
(3) The conclusions of this paper provide information and suggestions for decision-makers to implement the transformation and upgrading of intelligent manufacturing technologies.

The rest of this article is as follows: Section 2 reviews the relevant literature related to the research question. Section 3 describes the research methods and data collection and analysis procedures. Section 4 analyzes the results obtained from the proposed framework. Section 5 discusses the research results and its significance. Finally, section 6 systematically summarizes the research, discusses the limitations and proposes future research directions.

## 2 Theoretical background

According to the objectives of this study, the literature background of this study is divided into three structured parts: the context of intelligent manufacturing technology under industry 5.0 and sustainable development, the key challenges and enablers of implementing intelligent manufacturing technology in the context of industry 5.0 and sustainable development, and MCDM application in industry 5.0.

### 2.1 Industry 5.0 and sustainability context

With the development of technologies such as cyber-physical systems and industrial internet, Industry 5.0 has made rapid progress, bringing about a significant increase in productivity [11]. Guided by the development elements of Industry 5.0 and the concept of sustainable development, people are gradually realizing that human creativity and intellectual capacity can significantly improve the efficiency of intelligent manufacturing processes [[6], [12]]. Simple, repetitive, and routine tasks are assigned to machines or intelligent robots to improve efficiency, while tasks requiring critical thinking are assigned to humans to control quality and direction [3]. The application of intelligent manufacturing technology in this new context can not only maintain or improve the original production efficiency of the manufacturing industry but also ensure the core needs of employees and their work well-being.

Industry 5.0 emphasizes the development of a circular economy and the reuse of energy, which differs from the benefit-oriented development model of Industry 4.0. Within the framework of Industry 5.0, enterprises not only pursue production efficiency and economic benefits but also pay more attention to the environmental impact of the production process and the sustainable use of resources. With the continuous influx of advanced technologies such as Cyber-Physical Systems (CPS), Information Technology (IT), and Industrial Internet of Things (IIoT) [13,14], from personalized customer demands to digital twin simulation design [15], and then to the flexibility and sustainability of supply chains [9], intelligent manufacturing improves resource utilization efficiency and effectively enhances the resilience of sustainable development through means such as real-time data analysis and automation control.

While the evolution of industrial revolutions has promoted the transformation and development of all subsystems of society, it is undeniable that such transformations and developments have also had an impact on human society, the environment, and the economy [16]. With issues such as social demands, environmental pollution, and resource wastage becoming more prominent, existing manufacturing paradigms are struggling to meet the needs of the current innovative society [17]. Industry 5.0, with its flexible, human-centric, and sustainability-centered development concept and vision, is considered an exemplar for achieving a more sustainable digital transformation [18]. To promote the sustainable development of enterprises, industries, and nations, the United Nations has proposed 17 development goals and a common development framework, calling for countries to jointly draw the blueprint for the future of the Earth [11,19]. Although there may be some overlap, these goals typically encompass three aspects: social sustainability, environmental sustainability, and economic sustainability.

Although there is extensive research on the application of Industry 5.0, how to apply Industry 5.0 to achieve sustainable development transformation in enterprises remains a major issue. From the perspective of sustainable development, the challenges and enablers of applying Industry 5.0 are still unclear, which is not conducive to enterprises identifying the obstacles and driving forces in the development and transformation process [17] Accordingly, this study will lay the foundation for understanding the relationship between Industry 5.0 and social, environmental, and economic sustainability.

### 2.2 Key challenges and enablers

As the main vision and driving force of the current industry change, intelligent manufacturing technology gradually optimizes the manufacturing process and realizes more efficient, accurate and personalized manufacturing under the background of continuous technological innovation [20]. However, when the world is facing various crises such as resources, sustainable development has become the common pursuit of the global manufacturing industry [7]. At the same time, unlike Industry 4.0, which pursues ultimate efficiency and productivity, the social value and ecological value pursued by Industry 5.0 coincide with the current sustainable development needs and are changing the production paradigm of the manufacturing industry [13].

There are some scholars have explored the challenges, obstacles or enablers of implementing industrial 5.0 related technologies in the manufacturing sector [[1], [21]].

From the perspective of social sustainability, the Industry 5.0 paradigm emphasizes people-centered, which can not only reduce the tedious, dirty and repetitive work of human employees, but also promote the cooperation between human and artificial intelligence in the manufacturing industry and optimize human well-being [5]. However, how to design an intelligent manufacturing environment that can ensure production performance while giving priority to human well-being is one of the important challenges in the implementation process [22].

From the perspective of environmental sustainability, Industry 5.0 attaches importance to ecological value, emphasizes the cultivation and promotion of green innovation ability, accelerates green and low-carbon transformation, and improves the digital green innovation performance of enterprises. As an important part of modern economic activities, digital technology is an important driving force for green technology innovation. However, how to use intelligent manufacturing technology to expand the circular economy and create ecological benefits still plagues the industry [23].

In summary, this study explores the challenges and enablers of manufacturing industry to implement intelligent manufacturing technology from three perspectives of social, environmental and economic sustainability under the impetus of Industry 5.0. The following keywords and Boolean operators were used for literature screening when reviewing literature: “challenges” or “obstacles” or “barriers” or “enablers” and “industry 5.0” and “intelligent manufacturing” or “smart manufacturing” and “sustainability” or “sustainable development”. The literature was selected from the Web of Science database for literature search, and the time range was selected from 2020 to 2023. As a result, a list of challenges and enablers of intelligent manufacturing technology from the perspective of industry 5.0 and sustainability is preliminarily obtained.

### 2.3 MCDM application in industry 5.0

Currently, many classical Multiple Criteria Decision Making (MCDM) methods are widely applied in research related to Industry 5.0. They provide effective tools for identifying key influencing factors, exploring mechanisms, and formulating strategies, demonstrating broad applicability.

The Fuzzy Analytic Hierarchy Process (FAHP), as an extension of the Analytic Hierarchy Process (AHP), is used to address the uncertainties and ambiguities in Industry 5.0, improving the accuracy and reliability of decision-making processes [24], the AHP method is difficult to apply in processing large amounts of information [25,26] The Technique for Order of Preference by Similarity to Ideal Solution (TOPSIS) method determines the optimal solution by considering the distances between each solution and the ideal solution, demonstrating strong practicality and operability. Researchers have utilized the TOPSIS method to investigate the value sequence of different Industry 5.0 technologies in the healthcare industry [27]. Nevertheless, the TOPSIS method cannot ascertain the interrelationships between different factors.

The Decision Making Trial and Evaluation Laboratory (DEMATEL) method, as an important component of Multiple Criteria Decision Making (MCDM), is commonly used to deeply understand the causal relationships between internal factors by analyzing and modeling them[28,29]. It plays a crucial role in evaluating influencing factors and decision planning processes. Due to the novelty of the industry 5.0, there is a limited number of participants with relevant experience in emerging economies. DEMATEL demonstrates unique value in analyzing the correlations between internal factors [10]. Scholars have applied the DEMATEL method to determine strategic paths for sustainable development in Industry 5.0 [9] and to explore key factors influencing employee green behavior [25]. The manufacturing industry, as a complex system involving multiple roles and processes, must clearly identify challenges and driving factors before implementing intelligent manufacturing technologies to facilitate effective transformation and upgrading of manufacturing enterprises. Consequently, the DEMATEL method aligns well with the objectives of this study.

Although the Decision Making Trial and Evaluation Laboratory (DEMATEL) method can capture the relationships between challenges and enablers [30,31], it fails to reflect the structural levels and basic element classifications of factors within a system [32]. Interpretive Structural Modeling (ISM), as a qualitative analysis method, is a means of understanding complex relationships and hierarchies among elements within a system and has been widely applied in many fields [25,28]. ISM can help researchers clarify the hierarchical structure of factors and develop effective decision-making solutions Dwivedi et al., 2023. The AISM method enhances the original ISM with the addition of an adversarial approach to achieve Pareto optimality [32]. Therefore, this study integrates the DEMATEL method with the AISM method to optimize the hierarchical relationships between challenges and enablers.

Matrix Impacts Cross-reference Multiplication Applied to a Classification (MICMAC) method can determine the attributes and decision criteria relevant to the decision problem. Through MICMAC analysis, researchers can identify factors that have a significant impact on the development of Industry 5.0, providing a scientific basis for decision-making. This method is often combined with the Interpretive Structural Modeling (ISM) method to determine the relationship between different factors [[29], [33]].

The integration of DEMATEL with AISM-MICMAC methods facilitates a systematic and comprehensive clarification of the challenges and enablers for the implementation of intelligent manufacturing technologies under the sustainable development context of Industry 5.0. It accurately explores the relationships between different factors, thereby providing decision-makers with effective recommendations for implementing intelligent manufacturing technologies, laying a solid foundation for data analysis.

## 3 Materials and methods

**Fig. 1:**
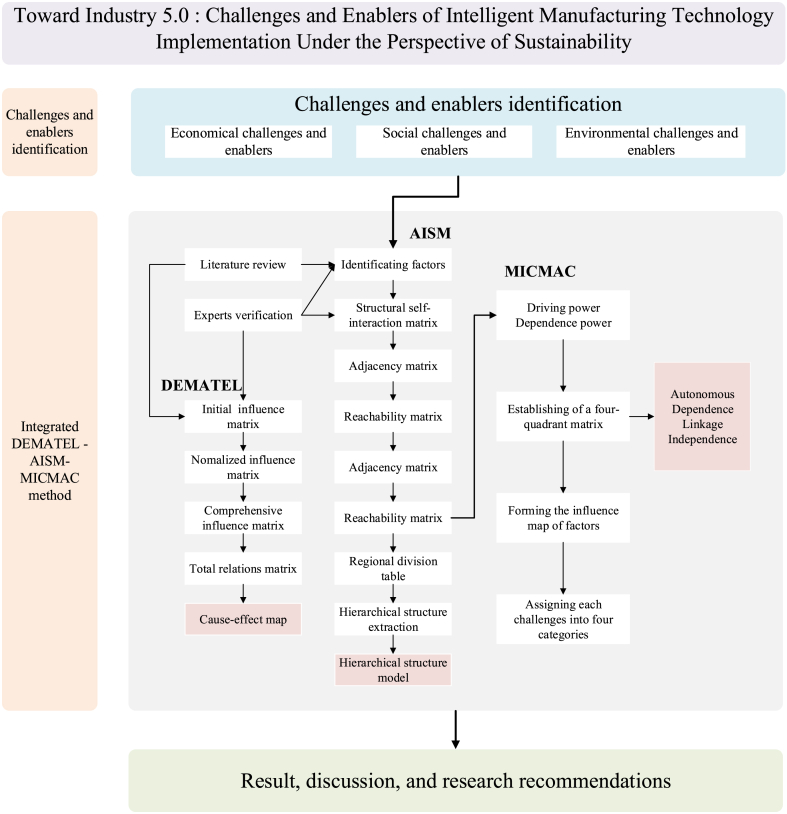
Proposed research methodology.

This study proposes a structured approach that combines DEMATEL and AISM-MICMAC analysis to identify key challenges and achievements in implementing intelligent manufacturing technologies under the context of Industry 5.0 and sustainable development. It determines the interrelationships, complexity, and impacts of factors, thereby promoting enterprises to achieve better development in a sustainable context. Fig. 1 depicts the essential key steps of the proposed research framework.

### 3.1 Sampling and data

The data for this study were collected and filtered in two phases. In the first phase, relevant literature was reviewed and integrated to extract potential challenges and enablers. Subsequently, a questionnaire was developed based on the sorted preliminary list of challenges and enablers. Using a snowball sampling method, 30 experts working in manufacturing enterprises or engaged in research related to the manufacturing industry were invited to participate in the survey anonymously through academic journals, conferences, and other channels. Finally, 15 key challenges and 15 key driving factors for the adoption of intelligent manufacturing technologies in the manufacturing industry under the context of Industry 5.0 and sustainable development were identified, as shown in Tables 1 and 2.

**Table 1:** Preliminary list of challenges from the literature review.

Sustainability	Code	Key challenges	References
Social	C1	Employment Pressure and Transformation	[7,34]
	C2	Legal and Regulatory	[3,10]
	C3	Social Acceptance and Adaptability	[10]
	C4	Data Security and Privacy Protection	[35]
	C5	Ethical and Social Issues	[22,36]
Environmental	C6	Total Energy Consumption	[37]
	C7	Total Carbon Emissions	[17]
	C8	Resource Reserve Pressure	[38]
	C9	Environmental Pollution	[38]
	C10	Ecosystem Stability	[7]
Economical	C11	Technology, Knowledge Absorption Pressure	[23]
	C12	Costs and Funding	[10]
	C13	Scalability	[3]
	C14	Supply Chain Management and Collaboration	[6,22]
	C15	Uncertainty of Market Competition and Business Environment	[37]

**Table 2:** Preliminary list of enablers from the literature review.

Sustainability	Code	Key enablers	References
Social	E1	Skills Upgraded and Employment Opportunity Provided	[39]
	E2	Work Environment and Welfare Improved	[22]
	E3	Social Benefits and Public Service Improved	[22]
	E4	Stakeholder Collaboration and Integration	[9]
	E5	Positive Organizational Culture and Values Influence	[40]
Environmental	E6	Energy Consumption Reducing	[3]
	E7	Carbon Emissions Reducing	[7]
	E8	Resource Utilization Efficiency Improved	[7]
	E9	Renewable Energy Integration	[[9], [38]]
	E10	Environmental Education and Awareness Raising	[41]
Economical	E11	Production Efficiency and Quality Improvement	[38,[42], [43], [44]]
	E12	Personalized Services and Products	[[9], [13]]
	E13	Resilience and Flexibility Enhancement	[45]
	E14	Supply Chain Management Informatization and Visualization	[37,42]
	E15	Payback Period Reduction	[45]

In the second data collection phase, the identified 15 key challenges and 15 key enablers were sent to six of the experts involved in the data survey in the first phase to solicit their opinions. Respondents will use a 6-point Likert scale ranging from 0 (very weak influence) to 5 (strong influence) to determine the magnitude of the relationship between challenge and enabler. The specific steps will be explained in the following sections.

### 3.2 Decision-making Trial and Evaluation Laboratory

Due to the complexity of the real environment, and the application of the DEMATEL method mainly depends on the intuitive perception of the evaluation experts on the influencing factors and the evaluation information given by their own experience and knowledge, it is very difficult to use accurate language to measure [46]. Therefore, this study introduces fuzzy language that is more in line with people 's way of thinking, closer to the actual situation, and can explain the evaluator 's point of view more conveniently and flexibly. The key steps of Fuzzy-DEMATEL in this study are described as follows.

Step1 Determining the correspondence between linguistic variables and fuzzy sets. The mapping relationship between linguistic variables and fuzzy sets is established, as shown in Table 3. And the expert 's scoring results of the influencing factors constitute the evaluation set. $X_{ij}$ denotes the influence degree of perception factor i on factor j based on expert k∈{1,2,…,n}. The number of factors analyzed is represented by n such that i,j∈{1,2,…,n}.

**Table 3:** Linguistic variable fuzzy set mapping table.

Language Variables	Separation System	Fuzzy Set Value Domain
No Effect (NO)	0	0
Very Low Impact (VL)	1	0∼0.25
Low Impact (L)	2	0.25–0.5
High Impact (H)	3	0.5–0.75
Very High Impact (VH)	4	0.75–1
Complete Impact (C)	5	1

Step2 Constructing a direct impact matrix between the relevant influencing factors of intelligent manufacturing technology. The individual evaluation matrix $X_{ij}$ is converted into a collective evaluation matrix by using the central ordered weighted average operator, and the collective evaluation matrix is converted into an accurate matrix A according to the distribution of linguistic variables and fuzzy sets, and the direct influence matrix A of DEMATEL is obtained.

Step3 Calculating the normalized matrix D.

$$D=A/b,A={\left(a_{ij}\right)}_{n\times n},b=\max_{1\leq i\leq n}\sum_{j=}^{n}a_{ij}$$

Step4 The normalized matrix D is used to calculate the combined influence matrix T. I is the unit matrix.

$$T=D{\left(I-D\right)}^{-1}$$

Step5 Calculating the causal relationship between the key factors: using the matrix D to calculate the value of the influence degree D, the affected degree C, the centrality M and the causality R.

$$D_{i}=\sum_{j=1}^{n}T_{ij},R_{j}=\sum_{i=1}^{n}T_{ij},i=1,2,\ldots ,n$$

### 3.3 Adversarial Interpretative structural modeling

This study combines the DEMATEL method with the AISM method to determine the mechanisms, hierarchical relationships, and functional paths of challenges and enablers in the implementation of intelligent manufacturing technologies under the context of Industry 5.0 and sustainable development. The key steps of AISM in this study are described as follows:

Step1**.** According to the combined influence matrix T obtained above, the overall influence matrix H is calculated, where T is the combined influence matrix and I is the unit matrix. The average value of all items in the combined influence matrix T is calculated and marked as λ.

H=T+I

Step2**.** The reachable matrix M is obtained by comparing the data in the global influence matrix H with λ.

$$M_{ij}\left\{ \begin{array}{c} 0\;if\;H_{ij}<\lambda \\ 1\;if\;H_{ij}<\lambda \end{array}\right.$$

Step3**.** After obtaining the reachable matrix M, the general skeleton matrix S is calculated by the formula.

$$S=M-{\left(M-I\right)}^{2}-I$$

Step4**.** The reachability matrix is decomposed by interval decomposition and inter-layer decomposition. The specific practice is to divide the factors into multiple levels according to the reachable set and the antecedent set, so that all the factors are divided into independent subsystems and divide the factors in the same system into different levels.

Step5**.** When obtaining the adversarial hierarchy graph, it is necessary to obtain the upward and downward topology levels at the same time. The upward topology hierarchy is also known as the result priority hierarchy extraction, which must follow the rule of extracting the final result factor first and then extracting the lower level factor in turn. The downward topology hierarchy is also known as the cause priority hierarchy extraction, which must follow the rule of extracting the fundamental factors first and then extracting the higher level factors in turn. Based on the hierarchical topology graph established by the skeleton matrix, the reachability relationship between the elements must be represented by directed line segments.

### 3.4 Matrix Impacts Cross-reference Multiplication Applied to a Classification

To further analyze the influencing factors, this study uses the MICMAC method to calculate the dependency and driving force of the factors, and to draw a four-quadrant diagram to identify different categories of factors in the system. The MICMAC method utilizes matrix multiplication principles to analyze the degree of influence and correlation between influencing factors by calculating the driving force (Q) and dependency (Y) of the factors. The greater the driving force, the more the factor helps in solving the given factor; the greater the dependency when solving the given factor, the greater the factor's dependency on other factors. The key steps of MICMAC in this study are described as follows:

Step1**.** According to the reachability matrix M, the driving force $DF\left(X_{i}\right)$ and dependence $DP\left(X_{i}\right)$ of each influencing factor are calculated.

$$DF\left(X_{i}\right)=\sum_{j=1}^{n}h_{ij}\left(i=1,2,\ldots ,n\right)$$

$$DP\left(X_{i}\right)=\sum_{i=1}^{n}h_{ij}\left(i=1,2,\ldots ,n\right)$$

Step2**.** According to the driving force and dependence data value of the influencing factors, the quadrant diagram is drawn. The mean value of driving force and dependence is regarded as the dividing line. As shown in the figure, the figure is divided into four quadrants: autonomy, dependence, correlation and driving.

Therefore, the influencing factors in the independent quadrant are more driven and less dependent on other factors, which proves that such factors are less affected by other factors but have a greater impact on other factors. Therefore, identifying such factors is of great significance for clarifying the process of intelligent manufacturing technology implementation in the context of industry 5.0 and sustainability.

## 4 Results

This section introduces the results of the implementation of the framework proposed above to identify, consider and evaluate the challenges and enablers of the arrival of Industry 5.0 for the implementation of intelligent manufacturing technology in the manufacturing industry. These challenges and enablers may hinder or promote the adoption of intelligent manufacturing technology in the manufacturing industry in the context of Industry 5.0.

**Fig. 2:**
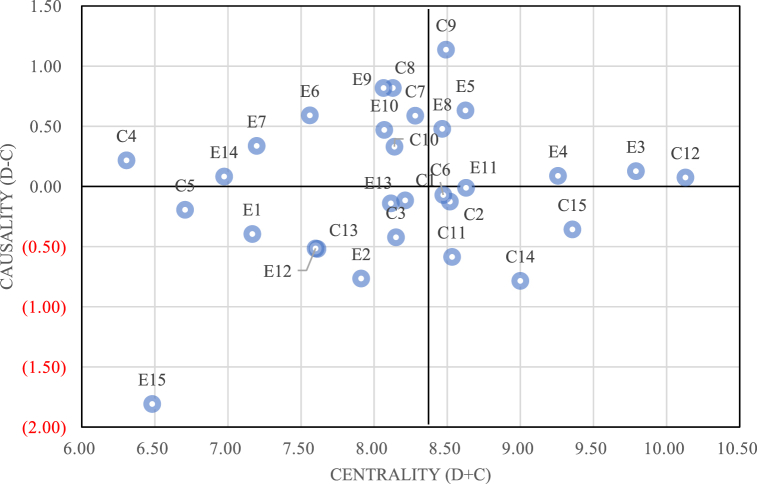
Centrality and causality.

After transforming the fuzzy matrix into an accurate matrix and generating the DEMATEL direct influence matrix based on the collective evaluation matrix, the influence matrix is generated according to the DEMATEL method and the DEMATEL analysis causality diagram is drawn, as shown in Fig. 2. In this figure, the centrality (D + C) is the x-axis, and the causality (D-C) is the y-axis. The 15 challenges and 15 enablers can be intuitively seen in the figure.

As shown in Fig. 2, according to the total effect matrix, the higher the centrality of the factor, the higher its importance. As shown in Fig. 5, the top ten rankings of challenge or enabler from large to small are: Costs and Funding (C12), Social Benefits and Public Service Improved (E3), Uncertainty of Market Competition and Business Environment (C15), Stakeholder Collaboration and Integration (E4), Supply Chain Management and Collaboration (C14), Production Efficiency and Quality Improvement (E11), Positive Organizational Culture and Values Influence (E5), Technology, Knowledge Absorption Pressure (C11), Legal and Regulatory (C2), Environmental Pollution (C9).

**Fig. 3:**
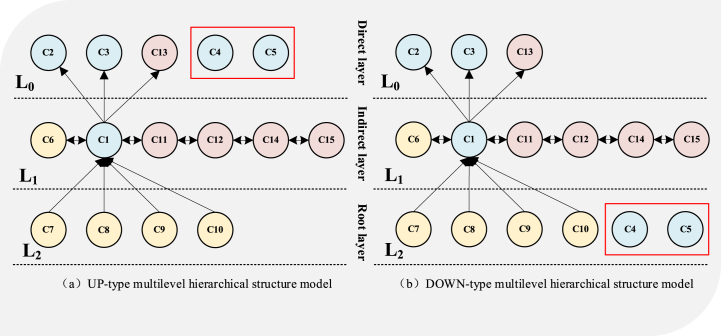
Key challenges hierarchy model.

**Fig. 4:**
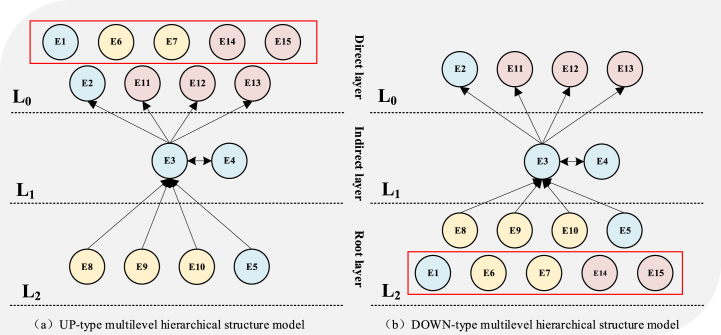
Key enablers hierarchy model.

After the combined influence matrix is obtained, the reachable matrix is obtained according to the steps in the previous 3.3 section to represent the influence relationship between all elements. After obtaining the reachable matrix, the reachable matrix M is decomposed between intervals and levels. The hierarchical structure of challenges and enablers constructed in this study is completed after three iterations, and the hierarchical structure diagram after iteration is shown in Fig. 3, Fig. 4.

**Fig. 5:**
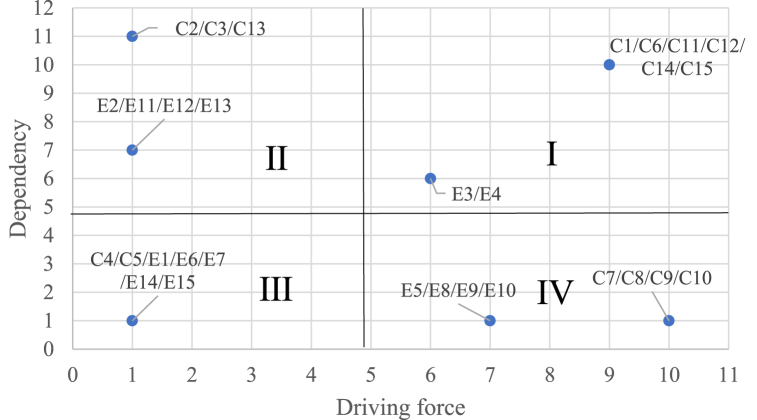
MICMAC analysis.

According to Fig. 3, apparently that most of the environmental sustainability challenges are located at the bottom of the system hierarchy and are the most fundamental and important aspects of the system 's key challenges; the challenges of economic sustainability are mostly located in the middle of the system hierarchy and are the indirect impact aspects of the system' s key challenge hierarchy. The challenges of social sustainability are mostly at the top of the system hierarchy, and most of them are direct influencing factors.

According to Fig. 4, compared with the key challenge hierarchy, in the key enabling hierarchy, the economic sustainability aspect at the top of the hierarchy is the direct influencing factor; the factors of environmental sustainability are distributed in the top structure and the bottom structure, which belong to the direct and fundamental influencing factors. The factors of social sustainability are distributed in three levels, and there is no obvious stratification.

The reachability matrix is used to calculate the driving force and dependence value of key challenges and enablers according to the steps described in Section 3.4, and the driving force and correlation value of each factor are filled as coordinates in the MICMAC analysis quadrant diagram. The average value of dependence and driving force is used as the boundary of the quadrant, and the key challenges and enablers factors are divided into four clusters: contact group (quadrant I), independent group (quadrant II), spontaneous group (quadrant III) and dependence group (quadrant IV). The division results are shown in Fig. 5.

The following part discusses the main findings and implications of this study.

## 5 Discussion

This study develops a structured framework for a comprehensive understanding of the challenges and enablers of intelligent manufacturing technology in the context of the implementation of Industry 5.0 in manufacturing from a sustainable perspective. Overall, the research results show that the smooth implementation of Industry 5.0 intelligent manufacturing technology requires a systematic and structured framework, so that enterprises and policy makers can collect valuable insights and suggestions from it, reasonably face up to challenges and take advantage of enablers.

According to the survey results of DEMATEL (Fig. 2), C12, C15, C14, C11 and C2 are the top five challenges in the importance of implementing industrial 5.0 intelligent manufacturing technology from a sustainable perspective. E3, E4, E11, E5 and E8 are the top five enablers in the importance of implementing industrial 5.0 intelligent manufacturing technology from a sustainable perspective. Among them, costs and funding (C12), social benefits and public service improved (E3) are the most significant challenges and the most important enablers for the implementation of intelligent manufacturing technology in the manufacturing industry under the research background[47,48]. Both of them are indirect influencing factors in the hierarchical structure model, and are in the contact group in the MICMAC analysis diagram. This shows that in the process of implementing intelligent manufacturing technology in the manufacturing industry under the sustainable background of industry 5.0, costs and funding have the most serious hindering effect, social benefits and public service improved have the most important promoting effect.

The AISM multi-level structural model in Fig. 3 indicates that the key challenge of environmental sustainability is a fundamental factor that directly or indirectly influences the challenges of economic sustainability and social sustainability. Carbon emission pressure (C7) [17] and environmental pollution pressure (C9) [48] may impose more constraints and challenges on manufacturing technologies for manufacturing enterprises. Policies and regulations may require the manufacturing industry to use cleaner energy and more environmentally friendly advanced manufacturing technologies. In the long run, although the introduction of green energy and new technologies can bring long-term economic benefits to enterprises, such as reducing management and operating costs [7], limited transformation investment forces manufacturing enterprises to face unfamiliar technologies and knowledge, and bear the pressure of absorbing and applying technologies and knowledge (C11) [23]. At the same time, the transformation of resources and technologies implies the re-establishment of the supply chain, forcing enterprises to face new supply chain management and coordination challenges (C14) [34]. The sustainable transformation of financial investment forces enterprises to face unknown and variable market competition and business environments (C15) [49]. Additionally, pressure on resource reserves (C8) may lead to higher costs and funds for procurement for manufacturing enterprises (C12), thus affecting the economic sustainability [50]. Furthermore, the rapid development of technology and the changing demand for knowledge (C11) may lead to changes in the demand structure of the labor market (C1), affecting employment pressure and labor force transformation [41,42].

As shown in Fig. 4, the schematic diagram of the multi-level hierarchical structure model of enabling factors indicates that certain factors of environmental sustainability are fundamental factors that directly or indirectly influence social and economic sustainability. Carbon reduction (E8), energy efficiency improvement (E9), and integration of renewable energy (E10) are three environmental enablers that prompt enterprises to reduce total energy consumption and save energy costsGhobakhloo, Iranmanesh, [9]. This means that manufacturing enterprises have more working capital available to strengthen their brand building, improve employee benefits (E3), support social welfare (E4), and so on. In today's environmentally conscious and socially equitable environment, enterprises and individuals increasingly emphasize the social benefits of their brands while ensuring product quality[51]. Manufacturing enterprises with high-quality social benefits are one of the reasons for market competitiveness. The positive brand effect is an inexhaustible driving force for attracting consumers and customers. Therefore, social sustainability factors can drive economic sustainability factors. When employee welfare (E3) and social benefits (E4) are positive, the enterprise's production efficiency and quality will be effectively improved (E11) [3,25]. It is also beneficial for providing personalized services and high-quality products to consumers (E12) [7]. Furthermore, it will enhance the resilience and flexibility of enterprises in facing market interventions or crises.

From the analysis results of MICMAC, it is known that the contact group factors in the first quadrant include C1, C6, E3, E4 and many other social and economic sustainability challenges or enablers. This group of factors has a high degree of driving force and dependence, and has a greater impact on the whole system. These factors will also be affected by other factors. Combined with the multi-level hierarchical structure diagram, it can also be seen that such factors are often located in the middle level of the hierarchical structure, which is in line with the actual situation. In the second quadrant, C2, C3, E2, E11 and many other challenges or enablers belong to the economic or social sustainability aspect. They are all located in the dependent cluster with high dependence and low driving force, and are often at the upper level of the multi-layer hierarchical structure model, which is also in line with the actual situation. The spontaneous factors in the third quadrant include many challenges and enablers such as C4, C5, E1, and E6. The driving force and dependence are low and relatively balanced, and play a transitional role in the system. From the multi-level hierarchical structure model diagram, those factors mostly exist in isolated factors, which is also in line with the actual situation. C7, C8, E5, E8 and other challenges and enablers in the fourth quadrant mostly belong to the environmental sustainability aspect, and all belong to the independent cluster with high driving force and low dependence. They are the driving factors in the multi-layer hierarchical structure, which are less affected by other factors. They are usually at the bottom of the model and belong to the root factors in the structural model, which will continue to have an impact on the system. This is also consistent with the analysis results of the DEMATEL method, which verifies the correctness of the method.

### 5.1 Managerial and policy implications

The analysis and findings of this study hold significant managerial and policy implications. The framework and conclusions proposed can assist manufacturing practitioners and policy makers in understanding the challenges and driving factors that the manufacturing industry may encounter when implementing or expanding intelligent manufacturing technologies within the context of Industry 5.0 sustainable development. Based on the afore mentioned results, this study puts forward the following suggestions.

(1) To overcome the barriers to the implementation of intelligent manufacturing technologies and enhance the positive impact on environmental sustainability development, manufacturing enterprises should pay attention to the challenges related to environmental sustainability. They should take proactive measures such as using clean energy and environmental protection technologies to reduce carbon emissions and environmental pollution.
(2) The lack of funds and financial resources is the biggest challenge leading to the slow progress of implementing intelligent manufacturing technologies. Therefore, enterprises need to increase investment in and research and development efforts for intelligent manufacturing technologies to enhance the production efficiency and quality of the manufacturing industry. Although transformational investments may face certain economic pressures, in the long run, the introduction of green energy and new technologies will bring long-term economic benefits to enterprises.
(3) Manufacturing enterprises should emphasize the management and coordination of the supply chain, which will help them address changes in resources and technologies, thereby enhancing the scalability and social adaptability of the enterprise.
(4) In terms of management decisions, enterprises should focus on the effective management of costs and funds to cope with the uncertainty of market competition and changes in the business environment. At the same time, enhancing employee welfare and improving social benefits will contribute to the improvement of production efficiency and the establishment of a good brand image for the enterprise.
(5) Government departments should strengthen policy support to provide more support and encouragement for the promotion of smart manufacturing technology. At the same time, promoting collaboration and integration among stakeholders will collectively drive the widespread application and development of smart manufacturing technology in the manufacturing industry.

The insights gained from this study can help policymakers and business decision-makers better address challenges and formulate long-term and short-term strategic decisions using driving factors, thereby strengthening the industry's forward-thinking, overall planning, and strategic layout. This ultimately achieves sustainable development, industrial technological transformation, and optimization. It can help managers and decision-makers understand the actual situation, thereby improving management efficiency in the implementation of functions such as management decision-making, and accelerating the technological revolution process of Industry 5.0.

### 5.2 Theoretical implications

This study provides some ideas for the promotion of Industry 5.0 and the sustainable development of manufacturing industry. First of all, this study focuses on the development direction of the manufacturing industry in the context of Industry 5.0, and establishes a clear link between the application expansion of intelligent manufacturing technology and the challenges and enablers of manufacturing sustainability. In the existing research, there is no research to explore this kind of relationship. Secondly, previous studies have not explored the challenges and enablers of social, economic and environmental sustainable development from the perspective of sustainability. This study attempts to use the MCDM method to explore the correlation and importance ranking between these challenges and enablers, and to fill this research gap through top-down and bottom-up hierarchical modeling analysis to verify the relationship between them. In addition, this study also provides a reference for identifying and exploring the literature research on the challenges and enablers of manufacturing industry to implement intelligent manufacturing technology in the context of Industry 5.0. Finally, based on the MCDM method, this study developed a framework that integrates DEMATEL and AISM-MICMAC models to identify, rank and reveal the relationship between challenge and enablers.

### 5.3 Case study

Industry 5.0 is not only a continuation and enhancement of Industry 4.0 but also a profound integration of human wisdom and advanced technology to achieve personalized customization, flexible production, and sustainable development. Advanced intelligent manufacturing systems, connected by cutting-edge technologies such as artificial intelligence (AI), the Internet of Things (IoT), cyber-physical systems (CPS), and digital twins (DT), are emerging one after another [13], [52]. Systems such as autonomous intelligent systems (AIS), autonomous intelligent manufacturing systems (AIMS), and human-information-physical systems (HCPS) have gradually constructed the industrial metaverse [[44], [51], [53]].

Among these, the human-information fusion intelligent manufacturing paradigm, exemplified by HCPS, has become a critical approach to addressing the complexity and uncertainty of manufacturing systems. By integrating human intelligence, information systems, and physical equipment, HCPS constructs a highly adaptive and dynamically responsive autonomous intelligent manufacturing system. This system enables deep integration and collaborative decision-making between humans and machines [54]. HCPS optimizes the production process, improves efficiency and quality, enhances the working environment, reduces labor intensity, and improves both work safety and employee satisfaction[43]. This aligns closely with the concept of human-machine collaboration in the Industry 5.0 transformation. 10.13039/100014337Furthermore, HCPS technology supports green manufacturing and sustainable development by optimizing energy management and resource utilization, thereby advancing the manufacturing industry towards environmental protection.

Additionally, the AIMS system, which integrates emerging technologies such as industry-GPT, DT, and knowledge graph (KG), boasts independent perception, cognition, and decision-making capabilities[44,55]. It effectively leverages the advantages of HCPS to promote the efficient, stable, safe, and low-carbon green operation of the manufacturing industry. The robust natural language processing capabilities of Industry-GPT provide AIMS with a deep understanding and analysis of complex production data. Real-time synchronization between the virtual DT model and the physical system allows AIMS to accurately simulate and predict manufacturing processes. By relying on these advanced information technologies, AIMS achieves a deeper integration of human-machine collaboration, enhancing the flexibility and adaptability of manufacturing systems[44]. Moreover, efficient energy management and resource utilization within AIMS facilitate the achievement of green manufacturing goals.

The development and expansion of the industrial metaverse have propelled the realization of the Industry 5.0 vision. This vision aims not only to improve efficiency and productivity but also to enhance the positive impact and contribution of industry and intelligent manufacturing to society and the environment. It reflects the characteristics of intelligence, flexibility, greenness, sustainability, and a people-oriented approach. Based on the significant achievements of the aforementioned intelligent manufacturing technologies, the evidence strongly supports the effectiveness and feasibility of the framework proposed in this study.

## 6 Conclusions

With the arrival of a new round of global scientific and technological revolution and industrial transformation, the era of industrial 5.0 is gradually approaching. Green, intelligent, environmentally friendly, high-quality development and industrial transformation have gradually become the main theme of the manufacturing industry. Although China 's intelligent manufacturing development has made great progress in recent years, problems such as low supply adaptability, low innovation ability and insufficient application depth and breadth still exist in the current development of intelligent manufacturing technology in manufacturing industry. Therefore, in order to realize the sustainable development of manufacturing society, economy and environment in the development of Industry 5.0, enterprises and political decision makers should promote the fundamental transformation of manufacturing industry mode and enterprise form by broadening the application scope of intelligent manufacturing technology and deepening the application depth of intelligent manufacturing technology. Firstly, according to the results of literature review and expert feedback, this study initially identified 15 key challenges and enablers, and used the DEMATEL method to analyze the above factors, and then used the AISM and MICMAC methods to further explore.

The analysis results of DEMATEL show that cost and capital, supply chain management and collaboration, scalability, ecosystem stability and laws and regulations are the key challenges that most seriously hinder the implementation of intelligent manufacturing technology in manufacturing industry. Social benefits and public service improvement, stakeholder collaboration and integration, production efficiency and quality improvement, positive organizational culture and value influence, and resource utilization efficiency improvement are the key enablers that most effectively promote the adoption of intelligent manufacturing technology in manufacturing. In contrast, the survey results of AISM and MICMAC show that four environmental sustainability challenges are the factors that most hinder the implementation of intelligent manufacturing technology in manufacturing industry, and three environmental sustainability enablers and social sustainability Positive organizational culture and values influence are the key factors that most strongly promote the application of intelligent manufacturing technology in manufacturing industry.

The innovation of this study is to systematically explore the key challenges and enablers of manufacturing industry to implement Industry 5.0 technology-intelligent manufacturing technology, explore different factors from the perspective of social, environmental and economic sustainability, and for the first time use fuzzy DEMATEL and AISM-MICMAC methods to identify, rank and reveal the links between different factors.

The contribution of this study is to provide strategic decision-making recommendations for business managers and policy makers to help them determine the challenges and enablers faced by the manufacturing industry in implementing industrial 5.0 intelligent manufacturing technology and their relationship in the context of sustainable development, which will help decision makers formulate short-term and long-term strategies. The above findings are of great significance for promoting the development of intelligent manufacturing and the technological change and optimization and upgrading of manufacturing industry.

Nevertheless, there are still some unsolved limitations in this study. First of all, although this study has obtained relevant challenges and enabling factors through comprehensive literature review and expert review, it may not be fully covered in some aspects. Secondly, the original data used in this study are entirely from the subjective evaluation data of Chinese professionals or industry experts. Therefore, the survey results may not fully reflect the reality, and there is a certain deviation from the actual situation. In the future research, the structural equation model (SEM) research conclusions may be used for verification. Finally, this study focuses on the strategy and decision-making of implementing intelligent manufacturing technology with manufacturing industry. In order to make the research conclusions more applicable, more data can be added in future analysis.

## Ethics statement

The authors get an approval of [Ethics Committee of Life Sciences of Zhengzhou University] to ensure that the research project complies with ethical standards, and the ethics approval number is IRB00006861. The authors confirm that informed consent was obtained from all participants in this study.

## Interest statement

The authors declare that they have no known competing financial interests or personal relationships that could have appeared to influence the work reported in this paper.

## CRediT authorship contribution statement

**Shiyan Liu:** Funding acquisition. **Pengyue Li:** Writing – original draft. **Jinfeng Wang:** Resources, Methodology. **Peng Liu:** Writing – review & editing.

## Declaration of competing interest

The authors declare that they have no known competing financial interests or personal relationships that could have appeared to influence the work reported in this paper.

## References

[1] Jwo J.-S., Lin C.-S., Lee C.-H. (2021). Smart technology–driven aspects for human-in-the-loop smart manufacturing. Int. J. Adv. Des. Manuf. Technol..

[2] Lu Y. (2021). The current status and developing trends of industry 4.0: a review. Inf. Syst. Front.

[3] Maddikunta P.K.R., Pham Q.-V., B P., Deepa N., Dev K., Gadekallu T.R., Ruby R., Liyanage M. (2022). Industry 5.0: a survey on enabling technologies and potential applications. Journal of Industrial Information Integration.

[4] Jagatheesaperumal S.K., Rahouti M., Ahmad K., Al-Fuqaha A., Guizani M. (2022). The duo of artificial intelligence and big data for industry 4.0: applications, techniques, challenges, and future research directions. IEEE Internet Things J..

[5] Johri P., Singh J.N., Sharma A., Rastogi D. (2021). Sustainability of Coexistence of Humans and Machines: an Evolution of Industry 5.0 from Industry 4.0 2021 10th International Conference on System Modeling & Advancement in Research Trends (SMART).

[6] Leng J., Sha W., Wang B., Zheng P., Zhuang C., Liu Q., Wuest T., Mourtzis D., Wang L. (2022). Industry 5.0: prospect and retrospect. J. Manuf. Syst..

[7] Iqbal M., Lee C.K.M., Ren J.Z. (2022). Industry 5.0: from Manufacturing Industry to Sustainable Society 2022 IEEE International Conference on Industrial Engineering and Engineering Management (IEEM).

[8] Ivanov D. (2022). The Industry 5.0 framework: viability-based integration of the resilience, sustainability, and human-centricity perspectives. Int. J. Prod. Res..

[9] Ghobakhloo M., Iranmanesh M., Morales M.E., Nilashi M., Amran A. (2022). Actions and approaches for enabling Industry 5.0‐driven sustainable industrial transformation: a strategy roadmap. Corp. Soc. Responsib. Environ. Manag..

[10] Mukherjee A.A., Raj A., Aggarwal S. (2023). Identification of barriers and their mitigation strategies for industry 5.0 implementation in emerging economies. Int. J. Prod. Econ..

[11] Bai C., Dallasega P., Orzes G., Sarkis J. (2020). Industry 4.0 technologies assessment: a sustainability perspective. Int. J. Prod. Econ..

[12] Gürdür Broo D., Kaynak O., Sait S.M. (2022). Rethinking engineering education at the age of industry 5.0. Journal of Industrial Information Integration.

[13] Javaid M., Haleem A. (2020). Critical components of industry 5.0 towards a successful adoption in the field of manufacturing. Journal of Industrial Integration and Management.

[14] Xiong G., Tamir T.S., Shen Z., Shang X., Wu H., Wang F.-Y. (2023). A survey on social manufacturing: a paradigm shift for smart prosumers. IEEE Transactions on Computational Social Systems.

[15] Lv Z. (2023).

[16] Tlili A., Huang R., Kinshuk (2023). Metaverse for climbing the ladder toward ‘industry 5.0’ and ‘society 5.0’?. Serv. Ind. J..

[17] Hein-Pensel F., Winkler H., Brückner A., Wölke M., Jabs I., Mayan I.J., Kirschenbaum A., Friedrich J., Zinke-Wehlmann C. (2023). Maturity assessment for Industry 5.0: a review of existing maturity models. J. Manuf. Syst..

[18] Zizic M.C., Mladineo M., Gjeldum N., Celent L. (2022). From industry 4.0 towards industry 5.0: a review and analysis of paradigm shift for the people, organization and technology. Energies.

[19] Carlsen L., Bruggemann R. (2021). The 17 United Nations' sustainable development goals: a status by 2020. Int. J. Sustain. Dev. World Ecol..

[20] Noor A.R.M., John J., Firyaguna F., Sherazi H.H.R., Kushch S., Vijayan A., O'Connell E., Pesch D., O'Flynn B., O'Brien W., Hayes M., Armstrong E. (2022). Wireless communications for smart manufacturing and industrial IoT: existing technologies, 5G and beyond. Sensors.

[21] Xiong G., Tamir T.S., Shen Z., Shang X., Wu H., Wang F.-Y. (2022). A survey on social manufacturing: a paradigm shift for smart prosumers. IEEE Transactions on Computational Social Systems.

[22] Coronado E., Kiyokawa T., Ricardez G.A.G., Ramirez-Alpizar I.G., Venture G., Yamanobe N. (2022). Evaluating quality in human-robot interaction: a systematic search and classification of performance and human-centered factors, measures and metrics towards an industry 5.0. J. Manuf. Syst..

[23] Yin S., Yu Y. (2022). An adoption-implementation framework of digital green knowledge to improve the performance of digital green innovation practices for industry 5.0. J. Clean. Prod..

[24] Bhyan P., Shrivastava B., Kumar N. (2023). Allocating weightage to sustainability criteria's for performance assessment of group housing developments: using fuzzy analytic hierarchy process. J. Build. Eng..

[25] Feng X., Li E., Li J., Wei C. (2023). Critical influencing factors of employees' green behavior: three-stage hybrid fuzzy DEMATEL–ISM–MICMAC approach. Environ. Dev. Sustain..

[26] Zientara P., Zamojska A. (2016). Green organizational climates and employee pro-environmental behaviour in the hotel industry. J. Sustain. Tourism.

[27] Abdel-Basset M., Mohamed R., Chang V. (2024). A multi-criteria decision-making framework to evaluate the impact of industry 5.0 technologies: case study, lessons learned, challenges and future directions. Inf. Syst. Front.

[28] Jain V., Qureshi H. (2022). Modelling the factors affecting Quality of Life among Indian police officers: a novel ISM and DEMATEL approach. Saf Health Work.

[29] Zhu X., Liang Y., Xiao Y., Xiao G., Deng X. (2023). Identification of key brittleness factors for the lean–green manufacturing system in a manufacturing company in the context of industry 4.0, based on the DEMATEL-ISM-MICMAC method. Processes.

[30] Su M., Woo S.H., Chen X., Park K.s. (2022). Identifying critical success factors for the agri‐food cold chain's sustainable development: when the strategy system comes into play. Bus. Strat. Environ..

[31] Yang J., Luo B., Zhao C., Zhang H. (2022). Artificial intelligence healthcare service resources adoption by medical institutions based on TOE framework. Digit Health.

[32] Chen H., Liu S., Wanyan X., Pang L., Dang Y., Zhu K., Yu X. (2023). Influencing factors of novice pilot SA based on DEMATEL-AISM method: from pilots' view. Heliyon.

[33] Usmani M.S., Wang J., Waqas M., Iqbal M. (2023). Identification and ranking of enablers to green technology adoption for manufacturing firms using an ISM-MICMAC approach. Environ. Sci. Pollut. Res. Int..

[34] Karmaker C.L., Bari A.B.M.M., Anam M.Z., Ahmed T., Ali S.M., de Jesus Pacheco D.A., Moktadir M.A. (2023). Industry 5.0 challenges for post-pandemic supply chain sustainability in an emerging economy. Int. J. Prod. Econ..

[35] Mourtzis D., Panopoulos N., Angelopoulos J., Wang B., Wang L. (2022). Human centric platforms for personalized value creation in metaverse. J. Manuf. Syst..

[36] Plumwongrot P., Pholphirul P. (2022). Are Robots stealing jobs? Empirical evidence from 10 developing countries. Econ. Innovat. N. Technol..

[37] Ahmed T., Karmaker C.L., Nasir S.B., Moktadir M.A., Paul S.K. (2023). Modeling the artificial intelligence-based imperatives of industry 5.0 towards resilient supply chains: a post-COVID-19 pandemic perspective. Comput. Ind. Eng..

[38] Sindhwani R., Afridi S., Kumar A., Banaitis A., Luthra S., Singh P.L. (2022). Can industry 5.0 revolutionize the wave of resilience and social value creation? A multi-criteria framework to analyze enablers. Technol. Soc..

[39] Verma A., Bhattacharya P., Madhani N., Trivedi C., Bhushan B., Tanwar S., Sharma G., Bokoro P.N., Sharma R. (2022). Blockchain for industry 5.0: vision, opportunities, key enablers, and future directions. IEEE Access.

[40] Broccardo L., Vola P., Zicari A., Alshibani S.M. (2023). Contingency-based analysis of the drivers and obstacles to a successful sustainable business model: seeking the uncaptured value. Technol. Forecast. Soc. Change.

[41] Grabowska S., Saniuk S., Gajdzik B. (2022). Industry 5.0: improving humanization and sustainability of Industry 4.0. Scientometrics.

[42] Adel A. (2022). Future of industry 5.0 in society: human-centric solutions, challenges and prospective research areas. J. Cloud Comput..

[43] Liu Q., Liu M., Zhou H., Yan F., Ma Y., Shen W. (2022). Intelligent manufacturing system with human-cyber-physical fusion and collaboration for process fine control. J. Manuf. Syst..

[44] Wang H., Wang C., Liu Q., Zhang X., Liu M., Ma Y., Yan F., Shen W. (2024). A data and knowledge driven autonomous intelligent manufacturing system for intelligent factories. J. Manuf. Syst..

[45] Yuan C., Liu W., Zhou G., Shi X., Long S., Chen Z., Yan X. (2022). Supply chain innovation announcements and shareholder value under industries 4.0 and 5.0: evidence from China. Ind. Manag. Data Syst..

[46] Feng X., Li E., Li J., Wei C. (2023). Critical influencing factors of employees' green behavior: three-stage hybrid fuzzy DEMATEL-ISM-MICMAC approach [Article; Early Access]. Environ. Dev. Sustain..

[47] Sharma M., Sehrawat R., Luthra S., Daim T., Bakry D. (2024). Moving towards industry 5.0 in the pharmaceutical manufacturing sector: challenges and solutions for Germany. IEEE Trans. Eng. Manag..

[48] Xu X., Lu Y., Vogel-Heuser B., Wang L. (2021). Industry 4.0 and industry 5.0—inception, conception and perception. J. Manuf. Syst..

[49] Ghobakhloo M., Iranmanesh M., Mubarak M.F., Mubarik M., Rejeb A., Nilashi M. (2022). Identifying industry 5.0 contributions to sustainable development: a strategy roadmap for delivering sustainability values. Sustain. Prod. Consum..

[50] Dwivedi A., Agrawal D., Jha A., Mathiyazhagan K. (2023). Studying the interactions among Industry 5.0 and circular supply chain: towards attaining sustainable development. Comput. Ind. Eng..

[51] Yao X., Ma N., Zhang J., Wang K., Yang E., Faccio M. (2022). Enhancing wisdom manufacturing as industrial metaverse for industry and society 5.0. J. Intell. Manuf..

[52] Fraga-Lamas P., Lopes S.I., Fernandez-Carames T.M. (2021). Green IoT and edge AI as key technological enablers for a sustainable digital transition towards a smart circular economy: an industry 5.0 Use case. Sensors.

[53] Cao L. (2022). Decentralized AI: edge intelligence and smart blockchain, metaverse, Web3, and DeSci. IEEE Intell. Syst..

[54] Wang B., Zheng P., Yin Y., Shih A., Wang L. (2022). Toward human-centric smart manufacturing: a human-cyber-physical systems (HCPS) perspective. J. Manuf. Syst..

[55] Zhou G., Zhang C., Li Z., Ding K., Wang C. (2019). Knowledge-driven digital twin manufacturing cell towards intelligent manufacturing. Int. J. Prod. Res..

